# *Actinobaculum schaalii*, a Common Uropathogen in Elderly Patients, Denmark

**DOI:** 10.3201/eid1601.090761

**Published:** 2010-01

**Authors:** Steffen Bank, Anders Jensen, Thomas M. Hansen, Karen M. Søby, Jørgen Prag

**Affiliations:** Viborg Hospital, Viborg, Denmark (S. Bank, T.M. Hansen, K.M. Søby, J. Prag); Aarhus University, Aarhus, Denmark (A. Jensen)

**Keywords:** Actinobaculum schaalii, real-time PCR, uropathogen, urinary tract infections, bacteria, research

## Abstract

*Actinobaculum schaalii* can cause urinary tract infections and septicemia but is difficult to identify by cultivation. To obtain a fast diagnosis and identify *A. schaalii*, we developed a TaqMan real-time quantitative PCR. Routine urine samples were obtained from 177 hospitalized patients and 75 outpatients in Viborg County, Denmark, in 2008–2009. The PCR detected *A. schaalii* in 22% of samples from patients >60 years of age. This assay showed that *A. schaalii* is more common than implied by routine cultivation. In 90% of PCR-positive urine samples, other common uropathogens were identified. This finding suggests that *A. schaalii* is a common, undetected, bacterial pathogen. Our results suggest that *A. schaalii* may be a more common pathogen than previously thought, especially in patients with unexplained chronic urinary tract infections, who are often treated with trimethoprim or ciprofloxacin, to which *A. schaalii* is resistant.

## Abstract 2

This organism is identified more often by PCR than by cultivation.

*Actinobaculum schaalii* was first described in 1997 and named after Klaus P. Schaalii, a German microbiologist specializing in actinomycete microbiology. The genus *Actinobaculum* includes *A*. *schaalii*, *A*. *suis*, *A*. *massiliae*, and *A*. *urinale* and is closely related to the genera *Actinomyces* and *Arcanobacterium* (*1*).

These bacteria are small, gram-positive, facultative anaerobic, CO_2_-requiring coccoid rods. They grow as dimorphic gray colonies <1 mm in diameter, are nonmotile and non–spore forming, and show weak β-hemolysis on agar plates containing 5% horse or sheep blood after 3–5 days of growth. They are catalase, oxidase, and urease negative and resistant to trimethoprim and ciprofloxacin (*2*). Their habitat is probably the human genital or urinary tract (*1*).

Because of its slow growth and resemblance to the normal bacterial flora on skin and mucosa, *A*. *schaalii* is often overlooked or considered a contaminant. Furthermore, it is often overgrown by faster-growing commensal and pathogen bacteria. Most laboratories incubate urine samples only overnight in ambient air, which further impedes isolation of *A*. *schaalii* (*2*).

Difficulties identifying *A*. *schaalii* by using traditional phenotypic tests have obscured its pathologic role for many years. However, *A*. *schaalii* can cause urinary tract infections (UTIs), some of which lead to serious illnesses such as urosepsis, osteomyelitis, and septicemia, mainly among the elderly and patients predisposed to UTIs (*1*–*6*). We developed a TaqMan real-time quantitative PCR (qPCR) specific for the gyrase B (*gyrB*) gene for fast and sensitive detection of *A*. *schaalii* from urine and blood samples.

## Materials and Methods

### Patient and Control Groups

From October 2008 through January 2009, a total of 252 routine urine samples were randomly selected from patients of all ages from 3 hospitals and 150 medical practitioners in Viborg County, Denmark (population ≈230,000 persons). Seventy percent of patients were from hospitals. Urine collection was midstream, from bedpans, from catheters, or unspecified in 41%, 19%, 18%, and 21% of cases, respectively. A total of 38 control urine samples were obtained from patients before they underwent elective surgery of hips or knees. These patients were 63–81 years of age and had negative results for leukocyte esterase and nitrate by a urine dipstick test (Roche Diagnostics Ltd., Burgess Hill, UK).

### Cultures and Wet Smear Microscopy of Urine Samples

Samples tested by using PCR were simultaneously analyzed by using standard laboratory tests. These tests were wet smear microscopy and incubation on 5% Columbia sheep blood agar (Becton Dickinson, Heidelberg, Germany) in an atmosphere of 5% CO_2_ at 35°C for 1 or 2 days.

### Extraction of DNA

Bacteria were incubated anaerobically on 5% Columbia sheep blood agar in an atmosphere of CO_2_ at 35°C for 2 days before harvesting. DNA was purified by taking a swab of bacteria from the agar plate and transferring it to 1 mL of saline. The DNA from bacteria was extracted from 800 μL of saline by using the Kingfisher mL magnetic particle processor (Thermo Electron Corporation, Waltham, MA, USA) according to the manufacturer’s instructions, eluted in 100 μL elution buffer, and stored at 4°C until use. DNA was also obtained from 800-μL urine samples as described above.

### Sequencing

Fourteen *A*. *schaalii* strains, including reference strain CCUG 27420, were used for sequencing. Universal primer pair UP-1 and UP-2r was used to amplify the *gyrB* gene from *A*. *schaalii* (Table 1). PCR was performed as described by Yamamoto and Harayama. (*7*). The PCR product was then gel purified by using the QIAquick Gel Extraction Kit (QIAGEN, Hilgen Germany) and sequenced in an ABI 3130 XL genetic analyzer (Applied Biosystems, Foster City, CA, USA) according to the manufacturers’ instructions. Sequencing primers UP-1S and UP-2Sr (Table 1) were used to sequence the purified PCR product in both directions. Primers were synthesized by DNA Technology (Aarhus, Denmark).

**Table 1:** Sequences of primers and probe used for identification of *Actinobaculum schaalii*, Denmark, 2008–2009

Primer or probe	Sequence (5′ → 3′)
UP-1	GAAGTCATCATGACCGTTCTGCAYGCNGGNGGNAARTTYGA
UP-2r	AGCAGGGTACGGATGTGCGAGCCRTCNACRTCNGCRTCNGTCAT
UP-1S	GAAGTCATCATGACCGTTCTGCA
UP-2Sr	AGCAGGGTACGGATGTGCGAGCC
A.s-forward	GGCCATGCAGTGGACCTC
A.s-reverse	GCACATCATCACCGGAAAGA
A.s-probe	TCCGAATCGGTCAATACCTTCGC

### Primers and Probe

Sequence alignment editor BioEdit (www.mbio.ncsu.edu/BioEdit/BioEdit.html) and Primer3 (http://frodo.wi.mit.edu/cgi-bin/primer3/primer3_www.cgi) programs were used to design a primer and probe specific for *A*. *schaalii* by multiple alignment of *gyrB* sequences from 14 *A*. *schaalii* strains, including reference strain CCUG 27420. Potential primers and probe were analyzed for the requirements imposed by real-time PCR by using PrimeQuest (http://eu.idtdna.com/analyzer/Applications/OligoAnalyzer/) and mfold (www.bioinfo.rpi.edu/applications/mfold/cgi-bin/dna-form1.cgi) programs. Selected primers and probe were analyzed for specificity against GenBank sequences by using the BLAST program (http://blast.ncbi.nlm.nih.gov/Blast.cgi).

The primer pair A.s-forward 5′-GGCCATGCAGTGGACCTC-3′ and A.s-reverse 5′-GCACATCATCACCGGAAAGA-3′ amplified a 185-bp fragment. The probe 5′-TCCGAATCGGTCAATACCTTCGC-3′ was labeled at the 5′ end with 6-carboxyfluorescein and at the 3′ end with Black Hole Quencher 1. Primers and probe were synthesized by Sigma-Aldrich (St. Louis, MO, USA).

### TaqMan qPCR

PCR amplification was performed by using a Mx3000P Real Time PCR System (Stratagene, La Jolla, CA, USA) in a 25-μL reaction volume. The PCR mixture contained 12 μL of 2× Brilliant QPCR Master Mixture (Stratagene), 2.5 μL of 100 nmol/L (final concentration) TaqMan probe, 2 μL of 200 nmol/L (final concentration) forward and reverse primers, and 5 μL of template DNA. An internal control containing 1.25 μL of internal PCR control primer/probe mixture and 0.25 μL of internal PCR control DNA (Applied Biosystems) was also used. Samples were incubated for 1 cycle at 95°C for 2 min and 50 cycles at 95°C for 30 s and 60°C for 60 s. All samples were run in duplicate. DNA from *A*. *schaalii* CCUG 27420 was used as a positive control and was included in each PCR. Sterile water was used as a negative control. Results were analyzed by using the Mx3000P software package (Stratagene).

### Detection Limit and Quantification

The detection limit of the *A*. *schaalii gyrB* assay was determined by using a 10-fold serial dilution of known concentrations (1.5 × 10^1^ to 1.5 × 10^8^ CFU/mL) of *A*. *schaalii* CCUG 27420. Quantification of *A*. *schaalii* in urine samples was performed by using the same dilution series.

### Analytical Specificity

To determine the analytical specificity of the assay, we tested 36 clinical strains of *A*. *schaalii* and strain CCUG 27420. Phylogenetically related (*1*) and clinically relevant bacterial strains, including several *Actinomyces* spp., *Arcanobacterium* spp., and reference strains *A*. *suis* CCUG 19026, *A*. *urinale* CCUG 46093, and *A*. *massiliae* CCUG 47753, were also tested (Table 2).

**Table 2:** Species used to test analytical specificity of gyrase B real-time PCR for *Actinobaculum schaalii*, Denmark, 2008–2009

Species	Source
*Actinobaculum* spp.	
*A. schaalii*	CCUG 27420*
*A. schaalii†*	Clinical isolates‡
*A. massiliae*	CCUG 47753
*A. suis*	CCUG 19026
*A. urinale*	CCUG 46093
*Actinomyces* spp.	
*A. gerencseriae*	Clinical isolates
*A. graevenizii*	Clinical isolates
*A. israelii*	Clinical isolates
*A. meyeri*	Clinical isolates
*A. naeslundii*	Clinical isolates
*A. neuii*	Clinical isolates
*A. odontolyticus*	Clinical isolates
*A. radingae*	Clinical isolates
*A. turicencis*	Clinical isolates
*A. urogenitalis*	Clinical isolates
*A. viscosus*	Clinical isolates
*Arcanobacterium* spp.	
*A. bernardiae*	Clinical isolates
*A. hemolyticum*	Clinical isolates
*A. pyogenes*	Clinical isolates
Other spp.	
*Gardnerella vaginalis*	Clinical isolates
*Rothia dentocariosa*	Clinical isolates
Common uropathogens	
*Alcaligenes faecalis*	Clinical isolates
*Candida albicans*	Clinical isolates
*Citrobacter koseri*	Clinical isolates
*Escherichia coli*	Clinical isolates
Hemolytic streptococcus group A	Clinical isolates
Hemolytic streptococcus group B	Clinical isolates
*Klebsiella oxytoca*	Clinical isolates
*K. pneumoniae*	Clinical isolates
Nonhemolytic streptococci	Clinical isolates
*Proteus mirabilis*	Clinical isolates
*Proteus vulgaris*	Clinical isolates
*Pseudomonas aeruginosa*	Clinical isolates
*Staphylococcus aureus*	Clinical isolates
*Staphylococcus epidermidis*	Clinical isolates

*GenBank accession no. FJ209064. †Thirty-six isolates. ‡GenBank accession nos. FJ518817–FJ518825.

### Verification of TaqMan qPCR Assay Results

To verify results of this assay, 6 PCR products were sequenced. The first 15 PCR-positive urine samples were cultivated, and isolates were identified as described by Reinhard et. al. (*2*). Identity of isolated *A schaalii* strains was confirmed by using a qPCR.

### Purification of DNA from Blood Cultures

Ten milliliters of blood and 1 mL of culture containing 2 × 10^7^, 2 × 10^5^, 2 × 10^3^, and 2 × 10^1^ CFU/mL of *A*. *schaalii* reference strain CCUG 27420 were added to aerobic and anaerobic BACTEC culture vials (Becton Dickinson). DNA from bacteria-positive blood cultures was extracted from 800 μL of aerobic or anaerobic media and purified by using the Kingfisher processor as described above.

Because BACTEC culture vials contain sodium polyanetholesulfonate (SPS), a known PCR inhibitor, either DNA must be purified from BACTEC culture vials by using specific purification methods or purified DNA must be diluted to prevent the SPS from inhibiting the PCR (*8*). Ten-fold serial dilutions of purified DNA from positive BACTEC culture vials were made and tested by using the qPCR as described above. DNA was extracted from an anaerobic BACTEC culture vial from a patient sample from which *A*. *schaalii* had been isolated by cultivation.

### Statistical Analysis

The χ^2^ test was used to analyze differences in detection of *A*. *schaalii*. Statistical analyses were performed by using SPSS for Windows version 16.0 (SPSS Inc., Chicago, IL, USA).

## Results

### Cultivation of PCR-Positive Samples

Isolates were obtained from 7 of the 15 urine samples cultured. The 7 isolates were confirmed positive by our real-time PCR.

### Detection Limit and Analytical Specificity

Assay results were linear at bacterial concentrations from 1.5 × 10^4^ to 1.5 × 10^8^ CFU/mL with an R^2^ value of 1.000 (Y = –3.296 × log(X) + 25.96). The detection limit of the assay was between 1.5 × 10^3^ and 1.5 × 10^4^ CFU/mL, which corresponds to 7.5–75 CFU/reaction. The assay amplified DNA from all 37 isolates of *A*. *schaalii* tested. No PCR amplification signal was detected when other species were tested (Table 2).

### DNA Sequencing Analysis

The 6 PCR products amplified from bacteria-positive urine samples had the expected size. Sequence alignment of the 6 PCR products showed homology to the sequenced *gyrB* gene from *A*. *schaalii* strains.

### Identification of *A*. *schaalii* from Blood Cultures

The 2 anaerobic BACTEC culture vials to which 1 mL of 2 × 10^7^ CFU/mL and 2 × 10^5^ CFU/mL had been added and 1 aerobic BACTEC culture vials to which 1 mL of 2 × 10^7^ CFU/mL had been added showed positive results in the BACTEC 9240 blood culture system. There was no growth recorded with lower inoculum concentrations.

PCR with undiluted and 10-fold diluted DNA was inhibited, probably by SPS. However, the 100-fold dilution of purified DNA from the 2 anaerobic and 1 aerobic BACTEC culture vials was PCR positive. The 100-fold dilution of purified DNA from a positive anaerobic BACTEC culture vial (patient specimen) was also PCR positive.

### Analysis of Urine Samples

Of 252 urine samples, 41 (16%) were PCR positive with bacterial concentrations >10^4^ CFU/mL. Of 155 urine samples from patients >60 years of age, 34 (22%) were PCR positive (Table 3), of which 31 (91%) harbored other common uropathogenic bacteria in addition to *A*. *schaalii* (Table 4). Species distribution of these common uropathogenic bacteria was comparable to that found in our microbiology department throughout the year. Treatment with antimicrobial drugs before specimens were obtained was reported by 19% of the patients.

**Table 3:** Distribution of *Actinobaculum schaalii* in 252 urine samples, Denmark, 2008–2009

Age of sample donors, y	No. (%) samples	95% CI	CFU/mL of *A. schaalii* in PCR-positive samples			
			10^4^–10^5^	>10^5^–10^6^	>10^6^–10^7^	>10^7^
0–10	12 (0)		0	0	0	0
11–20	16 (6)		0	0	0	1
21–30	21 (5)		1	0	0	0
31–40	15 (0)		0	0	0	0
41–50	11 (9)		1	0	0	0
51–60	22 (18)		2	2	0	0
61–70	52 (15)		4	3	0	1
71–80	54 (20)		4	4	1	2
>80	49 (31)		3	4	2	6
<60	97 (7)	3–14	4	2	0	1
>60	155 (22)	16–29	11	11	3	9
Healthy controls	38 (13)	4–28	2	3	0	0

*CI, confidence interval

**Table 4:** Uropathogens identified by cultivation of 155 urine samples from patients >60 y of age, Denmark, 2008–2009

Characteristic	*Actinobaculum schaalii*	
	PCR positive	PCR negative
Total no. samples	34	121
No growth	3	45
Uropathogens* >10^4^ CFU	31	76
*Escherichia coli*	13	38
Other *Enterobacteriaceae*	13	14
Other organisms		
Gram-negative aerobic rods	2	6
*Enterococcus faecalis*	0	6
Coagulase-negative staphylococci	0	4
*Aerococci* spp.	1	3
*Streptococcus* spp.	2	3
Yeast	0	2
>3 species	4	8

*Two species were identified in 4 of 34 PCR-positive samples and in 8 of 121 PCR-negative samples.

The 41 PCR-positive urine samples were collected midstream from 37% of patients, from bedpans for 27%, from catheters for 12%, and by an unspecified method for 24%. Among 177 hospitalized patients, 18% of samples from 104 patients >60 years of age and 10% of samples from 73 patients <60 years of age were PCR positive (p = 0.133). Among 75 urine samples obtained by practitioners, 30% of samples from 51 patients >60 years of age and none of the samples from 24 patients <60 years of age were PCR positive (p = 0.002). There was no significant difference in the presence of *A*. *schaalii* by sex of the patients (p = 0.485). When the control group (patients who had had hip or knee surgery) was compared with patients >60 years of age, no significant difference in the presence of *A*. *schaalii* was found (p = 0.227). In addition, we did not find any detectable differences between PCR-positive and PCR-negative results for hospitalized patients concerning underlying urinary tract pathologic changes and concurrent conditions such as hypertension and diabetes.

## Discussion

The real-time PCR assay confirmed that infection with *A*. *schaalii* increases with age (*2*). More than 1 of 5 urine samples from patients >60 years of age were PCR positive, and *A*. *schaalii* was most common in patients who visited medical practitioners and who had an infection with ordinary urinary pathogens. In comparison, culture findings in a study in our laboratory showed that 0.4% of cultured urine samples from patients >60 years of age had *A*. *schaalii* and that these patients had a broad spectrum of UTIs (*2*).

The present study shows that bacteria species, especially anaerobic or slow-growing species, are more common than what culture results indicate. Most likely, other pathogen bacteria exist that are even more difficult to identify by cultivation than is *A*. *schaalii*. Molecular biologic techniques such as real-time PCR can be valuable tools for identification of these organisms. Pathogenic bacteria that are difficult to cultivate or identify by cultivation should not be underestimated.

Other common uropathogens were identified by cultivation in 9 of 10 PCR-positive urine samples (Table 4). This finding indicates that *A*. *schaalii* is probably a common, undetected bacterial copathogen in many UTIs. Because most PCR-positive samples were from persons with multiple infections, determining which microorganism caused the UTI is difficult. However, results from our study support findings in case reports (*2*,*3*,*6*) in which *A*. *schaalii* was often found in monoculture for patients who had UTIs and therefore considered the causative agent. Furthermore, PCR showed that *A*. *schaalii* is a more common pathogen than previously thought. However, it will be difficult to fulfill the last of Koch’s criteria and prove with animal experiments that *A*. *schaalii* is a uropathogen.

Clinical microbiologists, clinicians, and medical practitioners should be aware of *A*. *schaalii* in patients predisposed for UTIs or unexplained chronic UTIs, especially if initial findings of wet smear microscopy for bacterial rods and leukocytes differ from negative growth under commonly used aerobic cultivation methods. For patients with suspected infections, urine should be sent to a department of clinical microbiology and incubated in an atmosphere of 5% CO_2_ for 2 to 3 days.

For patients with clinically verified UTIs who do not respond to treatment with ciprofloxacin or trimethoprim, infection with *A*. *schaalii* should be suspected. If *A*. *schaalii* is the cause of the infection, treatment with β-lactams, such as ampicillin or cephalosporins, should be given. The optimal duration of antimicrobial drug treatment with β-lactams is not clearly defined but several weeks of treatment may be required in severe cases.

Because *A*. *schaalii* can be difficult to identify even when cultured in an atmosphere of 5% CO_2_, the real-time PCR described in this report can be used for identification in urine and blood cultures. Alternatively, if the bacteria can be isolated by cultivation, the API Coryne and Rapid ID32A test systems (bioMérieux, Marcy l’Etoile, France) can be used for identification, as described by Reinhard et. al. (*2*). In conclusion, *A*. *schaalii* is an underestimated opportunistic copathogen that probably causes UTIs and urosepsis, particularly in elderly patients or patients predisposed for UTIs.
